# Lifetime stressor exposure is associated with altered trust formation and social learning in dynamic interactions

**DOI:** 10.1073/pnas.2608572123

**Published:** 2026-11-02

**Authors:** Jonas P. Nitschke, Nace Mikus, Livia Scanferla, George M. Slavich, Claus Lamm

**Affiliations:** ^a^ Department of Cognition, Emotion, and Methods in Psychology, Faculty of Psychology, University of Vienna, Vienna 1010, Austria; ^b^ Interacting Minds Centre, School of Communication and Culture, Aarhus University, Aarhus 8000, Denmark; ^c^Department of Neurology, Medical University Innsbruck, Innsbruck 6020, Austria; ^d^ Department of Psychiatry and Human Behavior, School of Medicine, University of California, Irvine, CA 92697-3950

**Keywords:** stress, trust, decision-making, social interactions, computational modelling

## Abstract

Although trust is fundamental to social interactions, how lifetime stressor exposure relates to the cognitive processes underlying trust remains poorly understood. Using the Stress and Adversity Inventory (STRAIN) to assess cumulative lifetime stressor exposure, combined with an adaptation of the repeated trust game and computational modeling (Hierarchical Gaussian Filtering), we investigated how stressors experienced over the life course relate to three core aspects of trust dynamics—initial trust formation, learning about others’ intentions, and adaptation to changes in trustworthiness—in 168 adults. Greater lifetime stress was associated with stronger negativity biases, lower initial trust, and heightened uncertainty about others’ intentions, increases that were general rather than specific to partner type. These more pessimistic priors were not accompanied by poorer reading of partners: Higher lifetime stress was associated with greater differentiation between prosocial and selfish trustees, driven by reduced investment in the selfish trustee alone. Computational analyses further indicated greater belief volatility, consistent with faster model-implied updating of perceived trustworthiness. These findings provide novel evidence that lifetime stress is associated with a profile consistent with recalibration, rather than a degradation, of the cognitive mechanisms underlying trust, with potential consequences for relationship formation and social functioning. They also provide a computationally grounded framework for understanding associations between adversity and social cognition and generate testable predictions for future experimental work.

## Significance

Lifetime stress may be linked to how individuals navigate social relationships and form beliefs about others’ trustworthiness. Using computational modeling of dynamic trust interactions, we find that lifetime stress is associated with amplified negativity biases, reduced initial trust, and heightened uncertainty about others’ intentions. Notably, these more negative expectations were not accompanied by poorer reading of partners: Higher-stress individuals distinguished trustworthy from untrustworthy partners more, not less, sharply. This pattern is consistent with recalibration, rather than degradation, of mechanisms underlying trust. This cross-sectional study thus provides a framework for understanding the role of adversity in social cognition and yields testable predictions for future longitudinal and causal research.

Social relationships are essential for health and well-being, with trust serving as a foundational component of functional social interactions (1, 2). Trust, however, is not static, but rather a dynamic process that is continually shaped by past experiences and real-time interactions. This adaptability renders trust particularly vulnerable to stress. According to Social Safety Theory (1–3), for example, cumulative exposure to social threat recalibrates how individuals appraise others’ intentions. According to this formulation, stress would be expected to bear specifically on trust. Yet, how stressors occurring across the life course relate to cognitive processes underlying trust remains untested—a gap that matters because stress-related changes in trust may weaken the very relationships that typically buffer against adverse outcomes. To investigate, we assessed lifetime stressor exposure using the gold-standard Stress and Adversity Inventory (STRAIN), a comprehensive measure of cumulative lifetime stressor exposure. In addition, we leveraged Hierarchical Gaussian Filtering within a dynamic trust game paradigm to quantify trial-by-trial fluctuations in three key cognitive parameters: belief volatility, negativity bias, and uncertainty. Doing so enabled us to examine how lifetime stress is related to three fundamental aspects of social trust–namely, trust formation, adaptive learning, and responses to trust violations.

Social relationships serve multiple critical functions, providing emotional support (2, 4), buffering against adverse events (5, 6), enabling collaboration (7), and ultimately improving survivability (for reviews: 8, 9). Furthermore, social relationships can buffer the negative effects of stress in both acute and more chronically stressful situations (10–13). These protective and adaptive effects depend fundamentally on a person’s ability to interpret others’ intentions and form accurate expectations about their future behavior—a process central to trust and cooperation. However, interpreting others’ intentions—even when prior knowledge exists—remains complex, and navigating our social environment is a highly demanding endeavor, particularly when information is limited or ambiguous (for a review: 14).

To manage this challenge, humans are intrinsically motivated to reduce uncertainties by actively engaging with our social and nonsocial environments: exploring actions, observing consequences, and updating expectations accordingly (for theory: 15). This capacity for social prediction enables humans to form and maintain stable, supportive relationships by fostering connections with individuals deemed trustworthy while avoiding those who demonstrate unreliable or selfish behaviors (for a review: 16). Predicting and monitoring whom to trust relies on fundamental processes linked to belief formation and updating. Initially, we establish probabilistic expectations about others’ intentions through repeated interactions. These beliefs then adapt as new information comes online—such as shifts in others’ behaviors—reflecting the dynamic nature as we learn from experience. Notably, these probabilistic expectations serve as prior expectations that guide subsequent social decisions. These expectations are continuously refined as new behavioral evidence accumulates, allowing trust to adapt to changing social contexts.

Experimental paradigms like the repeated version of the trust game can effectively capture this learning or updating process. This extension of the classical single-shot version of the trust game, extensively used in behavioral and neuroeconomics, requires investors to balance their prior expectations about social interactions against unfolding behavioral patterns across repeated interactions (17–20). Optimal performance in the game demands that participants not only adjust their strategies based on their interaction partner’s (“the trustee”) current behavior—whether to avoid exploitation or foster cooperation—but also monitor the social environment’s stability. This adaptive challenge is precisely what computational models formalize as environmental volatility: the degree to which an individual’s or environment’s actions fluctuate over time, requiring continuous recalibration of predictions and strategies (21, 22). The ability to accurately track and respond to such volatility determines whether individuals can maintain effective social strategies.

Yet, this adaptive capacity to navigate uncertainty and volatility appears vulnerable under various types of stress. For instance, stress and uncertainty are closely coupled: The experience of uncertainty has been shown to drive acute stress responses (23), a link likely to extend to ambiguous social contexts.

Because trust priors are built over a lifetime of social experience, cumulative stressor exposure—rather than current distress or dispositional stress susceptibility—is the theoretically matched predictor of how individuals appraise others (2, 24, 25). This reasoning also motivates distinguishing early-life from adulthood exposure, and thus asking whether the association with trust-related beliefs is carried by stressors from a single period or by their accumulation across the life course. Although research has shown that chronic stress can impair cognitive functioning and affect decision-making processes (26–29), we know of no studies that have investigated how stressors occurring over the entire life course relate to dynamic social interactions.

Several hypotheses are in need of exploration in this context. For instance, repeated stress exposure may prime an individual to expect high environmental volatility–that is, the belief that one’s immediate environment, including one’s social environment, is highly changeable (for a review: 30). Indeed, research has linked persistent stress exposure to heightened perceptions of uncertainty about the world and reduced optimism (31), which may extend to broader expectations about one’s environment—including social interactions and relationships. This heightened perception of instability may trigger excessive belief updates even in response to minor cues, potentially leading to overly cautious or impulsive decisions as individuals prioritize immediate information over established patterns. Supporting this possibility, Raio et al. (32) found that greater lifetime stressor exposure is associated with increased ambiguity aversion—the tendency to avoid decisions where the probabilities of outcomes are unknown—but not for risky choices in general. In social contexts, such ambiguity aversion could manifest as avoidance of uncertain social situations (e.g., when others’ intentions are unclear), resulting in heightened caution or withdrawal. Furthermore, in an exploratory individual-differences analysis, de Berker et al. (23) found that higher levels of perceived (chronic) stress were associated with a higher metavolatility parameter—a computational index of the belief that one’s environment is unstable, conceptually analogous to the volatility parameter (ω) we model here. Although this association stems from a nonsocial aversive learning task, it raises the possibility that stress is accompanied by a more general expectation of environmental volatility, which may extend to the social domain. Similarly, Soares et al. (33) found that higher levels of chronic stress were associated with changes in decision-making, particularly by shifting choices toward relying on habitual behaviors rather than flexible, goal-directed actions. This pattern is consistent with broader evidence that stress impairs cognitive flexibility—the ability to adaptively tailor responses to specific contexts (27, 32, 34–36).

In social contexts, this rigidity may lead to systematic misinterpretations of others’ intentions in fluid social environments. Moreover, stress induces a negativity bias—an increased expectation of negative over positive outcomes—that particularly affects social cognition (37–39). In trust-based interactions, this bias could result in lower expectations of reciprocal behavior and consequently reduced investments, even when cooperation would be mutually beneficial. Although research has demonstrated how acute stress impairs cognitive flexibility and decision-making (for reviews: 40–42), the effects of lifetime stressor exposure on social cognition remain unclear.

To address this critical gap, we leveraged a dynamic repeated trust game paradigm combined with computational modeling to investigate trust formation and adaptation across multiple interactions with partners exhibiting varying reciprocity. Although chronic stress is known to impair decision-making and cognitive flexibility, its real-time effects on social exchanges—particularly during dynamic shifts such as sudden betrayals after established cooperation—remain unexplored. Using the Stress and Adversity Inventory (STRAIN) (43) as a comprehensive measure of cumulative lifetime stressor exposure, we tested our primary hypothesis that greater lifetime adversity is associated with differences in trust decisions. In our novel paradigm, participants first learned the trustworthiness of partners before experiencing unexpected behavioral reversals, enabling us to assess how lifetime stress relates to adaptability under uncertainty. We then used Hierarchical Gaussian Filtering (HGF) (21, 22) to model trial-by-trial belief updates, characterizing the cognitive processes underlying both dynamic trust formation and continuous adaptations.

## Results

### Cumulative Lifetime Stressor Exposure.

Participants’ mean lifetime stressor severity in the present sample was 66.6 (SD = 37.1; range = 5 to 198), which corresponds to an average severity rating of moderate stress experience, and was comparable to the validation sample for the German-language version administered here (44).

### Behavior: Lifetime Stressor Exposure Is Related to Differential Levels of Investment in the Repeated Trust Game.

**Fig. 1.**
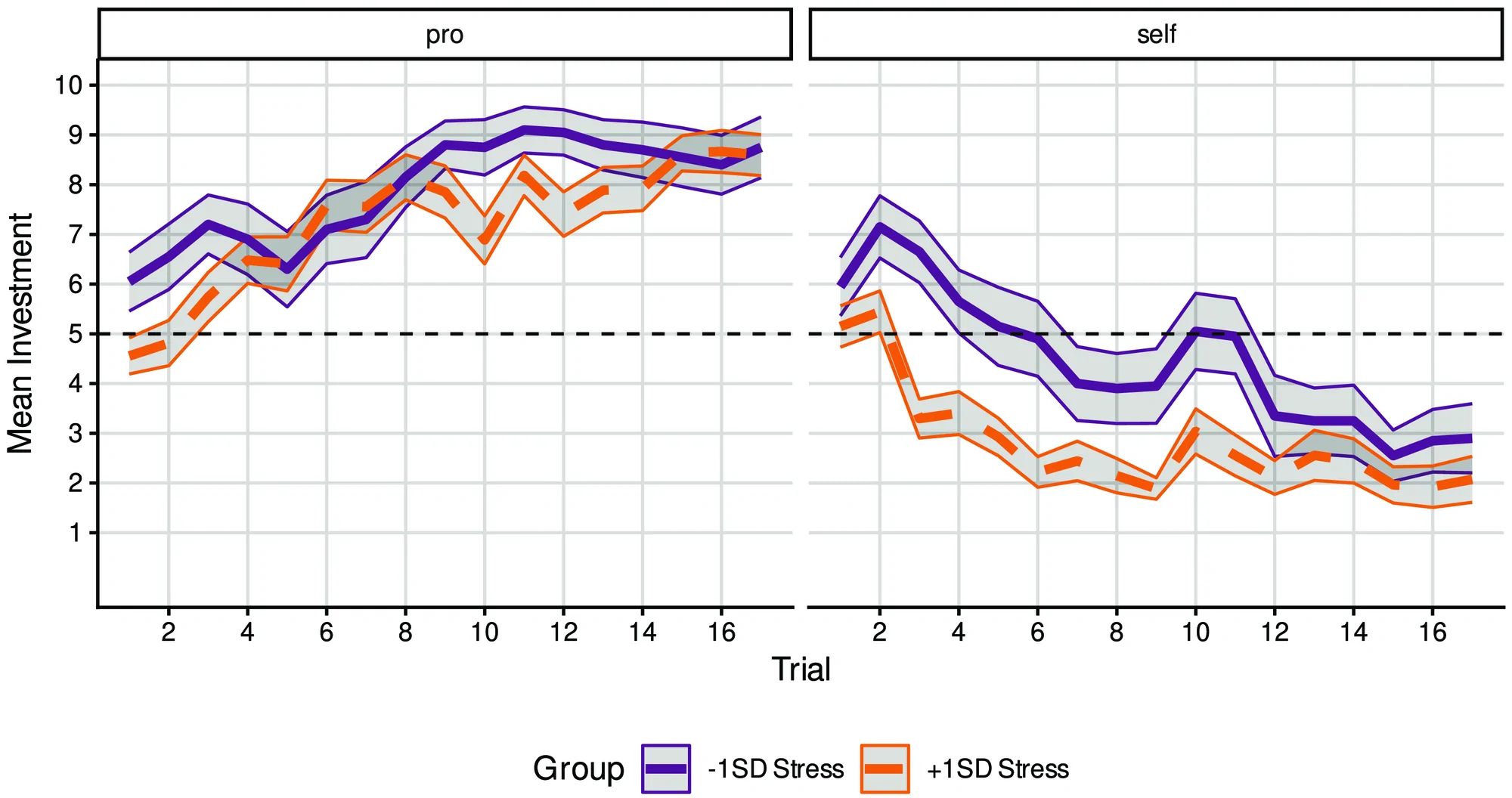
Mean investment for each trustee (*Left* panel: prosocial trustee; *Right* panel: selfish trustee) over the first 17 trials (learning phase) by lifetime stressor exposure levels (plotted as one SD below (solid, purple) and above (dashed, orange) the mean; for illustration purposes only, analyses conducted with continuous data; shaded bands represent ± SEM). Participants generally increased investment toward the prosocial trustee and decreased investment toward the selfish trustee across trials, reflecting successful differentiation between the two interaction partners. Critically, individuals with higher lifetime stressor exposure (dashed, orange) invested less in the selfish trustee compared to those with lower exposure (solid, purple), while investment in the prosocial trustee remained similar across stress levels, suggesting that lifetime stress is associated with heightened caution toward potentially exploitative partners rather than a generalized reduction in trust.

To test the association between lifetime stress and investment behavior in the trust game, we estimated a Bayesian mixed-effects model for the initial learning phase (2 × 17 trials). The model revealed two robust two-way interactions. The interaction for trustee × trials showed a robust effect (−0.399, CrI[−0.441, −0.359]) with the posterior probability indicating a compelling negative effect (P(Eμtrustee × trials < 0) > 0.999). This indicated that participants—throughout the first 34 trials—differentiated between the prosocial and the selfish player (for prosocial: 0.194, CrI[0.165, 0.224]; for selfish: −0.205, CrI[−0.237, −0.174]). In addition, there was a robust interaction for trustee × stress (−0.314, CrI[−0.562, −0.071]) with the posterior probability indicating a compelling negative effect [P(Eμtrustee × stress < 0) = 0.994], indicating that stress was differentially associated with investment behavior for prosocial and selfish actors—with post hoc tests revealing that greater lifetime stressor exposure was robustly associated with less investment to the selfish trustee (−0.482, CrI[−0.767, −0.206]), and no effect for the prosocial trustee (−0.167, CrI[−0.478, 0.141]; Fig. 1). Furthermore, we found no evidence that lifetime stress altered the rate of learning across trials (trial × stress: −0.007, CrI[−0.036, 0.021]), nor that this pattern differed between trustee types (trial × stress × trustee: 0.009, CrI[−0.031, 0.048]). These findings suggest that while lifetime stressor exposure is associated with the level of investment, it does not alter how investment behavior changes over the course of repeated interactions. See Table 1 for results from the Bayesian regression. Sensitivity analyses accounting for the feedback participants received are reported in *SI Appendix*, Table S7.

**Table 1.** Rank-ordered Bayesian regression predicting investment from lifetime stressor exposure, trustee type, and trial

	Estimate	low CrI	high CrI
Intercepts	“The full set of Intercept estimates is provided in *SI Appendix*, Table S1.”		
Age	0.001	−0.226	0.225
Gender (0 = men)	−0.417	−0.981	0.143
Trial	**0.194**	**0.165**	**0.224**
Trustee (0 = prosocial)	0.037	−0.203	0.277
Stress	−0.167	−0.478	0.141
Trial × Trustee	**−0.399**	**−0.441**	**−0.359**
Stress × Trustee	**−0.314**	**−0.562**	**−0.071**
Trial × Stress	−0.007	−0.036	0.021
Trial × Stress × Trustee	0.009	−0.031	0.048

Note. Fixed effects from the ordinal Bayesian regression on investment amount (rank-ordered; 1 to 10). Age and Gender are included as covariates. For the interaction partner (“trustee”), the reference group is set to the prosocial trustee. Stress refers to lifetime stressor exposure, operationalized as the z-standardized cumulative lifetime stressor severity score from the STRAIN. Boldface indicates coefficients with 95% credible intervals excluding zero.

### Modeling Parameters: Higher Lifetime Stressor Exposure Is Associated with Lower Trust Expectations and Greater Model-Implied Belief Volatility.

**Fig. 2.**
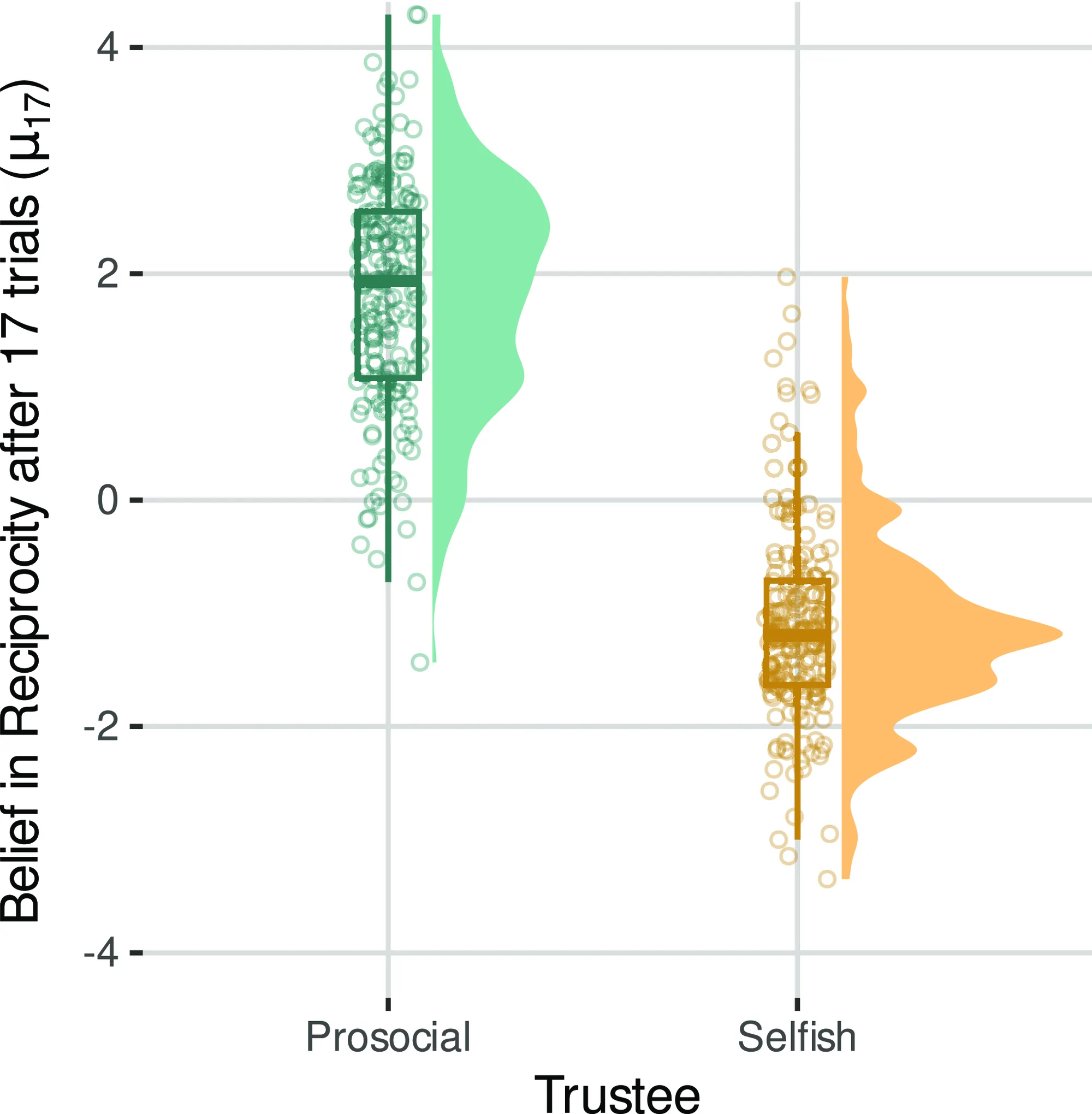
Belief in reciprocity at the end of the learning phase (μ_17_; i.e., after 17 trials each), shown separately for the prosocial and selfish trustee (n = 168). Open circles represent individual participants; boxplots show the median and interquartile range, with whiskers extending to 1.5 × IQR; density curves show the distribution of beliefs for each trustee. Beliefs separated clearly by trustee type, indicating that the model captured participants’ learned expectations and that the paradigm tracked trust formation trial by trial.

After establishing the associations of lifetime stress with investment behavior, we aimed to identify factors and computational processes underpinning these associations by using the HGF to infer the participants’ beliefs about the other trustee’s intentions. By the end of the learning phase, model-inferred beliefs separated clearly by trustee type, confirming that the model captured participants’ learned expectations (Fig. 2). We first correlated parameters derived from the best-performing HGF model with lifetime stressor severity, as assessed by the STRAIN and z-scaled, to probe stress-associated expectations about the trustees’ behaviors. Here, we found that greater lifetime stressor exposure was related to lower initial expectations of reciprocity (μ_0_), r = −0.161, *P* = 0.038, indicating that participants with greater stressor exposure entered the task with lower trust. Next, we found that the expectation bias (ρ) correlated negatively with lifetime stress, r = −0.279, *P* < 0.001, indicating that greater stressor exposure was associated with more negative expectations about reciprocity (i.e., a “negativity bias” toward trustees’ trustworthiness).

**Fig. 3.**
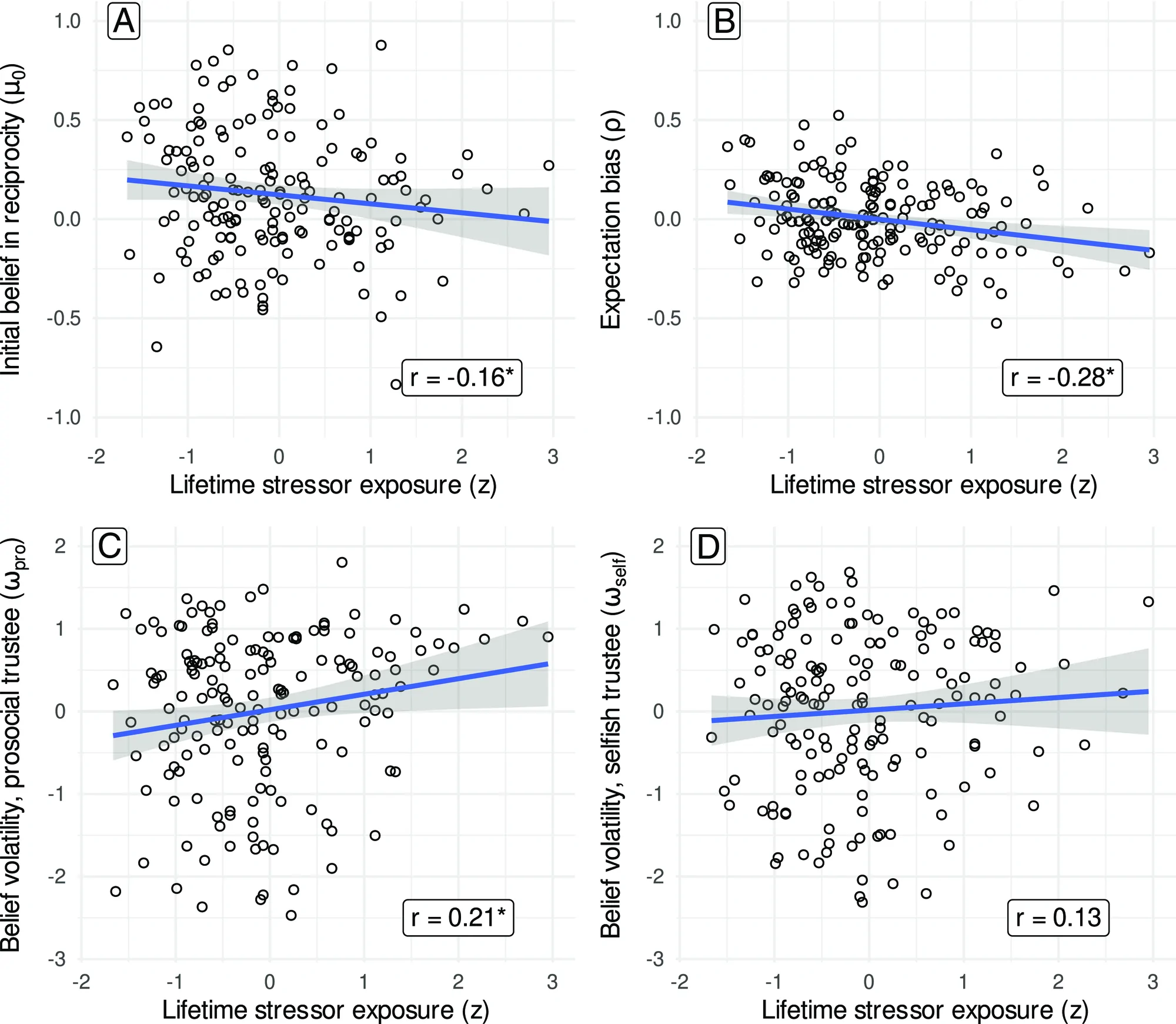
Correlations between z-scaled lifetime stressor exposure (*x*-axis) and belief parameters derived from the Hierarchical Gaussian Filter (*y*-axis): (*A*) initial belief in reciprocity (μ_0_); (*B*) expectation bias (ρ); (*C*) belief volatility for the prosocial trustee (ω_pro_); (*D*) belief volatility for the selfish trustee (ω_self_). Each panel shows individual participant data (open circles; n = 168) with a linear regression line and 95% confidence band, and the Pearson correlation coefficient. Volatility parameters (*C* and *D*) are shown z-scored. Higher values on the *x*-axis reflect greater cumulative lifetime stressor exposure. Greater lifetime stressor exposure was associated with lower initial expectations of reciprocity (*A*) and a stronger negativity bias toward trustees’ intentions (*B*), consistent with more pessimistic expectations at the outset of the interaction. Greater lifetime stressor exposure was also associated with more volatile beliefs about the prosocial trustee’s intentions (*C*), with no convincing association for the selfish trustee (*D*). *P < 0.05.

We then investigated how lifetime stressor exposure related to belief volatility ω. We found a significant association between stress and ω_average_ r = 0.185, *P* = 0.017, such that greater lifetime stressor exposure was associated with more volatility in beliefs about others’ intentions. This finding suggests that individuals with higher lifetime stress may perceive others’ behavior as more unpredictable and changeable. Next, we correlated ω separately for the type of trustee, for the trustee that starts as prosocial but becomes selfish after 17 trials (ω_pro_), and for the trustee that starts as selfish but turns prosocial (ω_self_). This analysis revealed a significant association for the prosocial trustee (ω_pro_), r = 0.207, *P* = 0.007, with no convincing evidence for a correlation with the selfish trustee (ω_self_), r = 0.127, *P* = 0.103 (Fig. 3). The two correlations did not differ significantly [t(165) = 1.09, *P* = 0.28]. The association was numerically strongest for ω_pro_, but we do not interpret it as partner-specific.

Because these parameters could reflect a single stress-related profile or separate components, we next examined their overlap. The negativity bias (ρ) and belief volatility (ωpro) were unrelated, both before and after removing shared stress-related variance, indicating separable components rather than one undifferentiated effect (*SI Appendix*, Table S6). Decomposing the associations further, lower initial trust (μ_0_) and the negativity bias (ρ) shared part of their association with lifetime stressor exposure, whereas belief volatility showed little comparable overlap (*SI Appendix*, Table S5). Because all three parameters are derived from the same behavioral data, this pattern reflects concurrent associations rather than evidence for a directional or mediating pathway.

Next, we examined whether the timing of stressor exposure (i.e., early life vs. adulthood) mattered. We found a significant association between expectation bias (ρ) and adulthood stressors (r = − 0.237, *P* < 0.001), but no conclusive evidence for an association with early-life stressors (r = −0.146, *P* = 0.059). No significant associations emerged for ω_pro_ or ω_self_, indicating that the observed association with belief volatility was evident only for cumulative lifetime stressor exposure. Thus, within the present sample, associations with trust-related belief parameters appeared more evident for cumulative and adulthood stressor exposure than for early-life stressors (see Table 2 for a summary). In summary, these results suggest that greater lifetime stressor exposure is associated with lower initial beliefs about trust, a more negative expectation bias regarding trustees’ reciprocity throughout the task, and greater belief volatility for the initially prosocial trustee, with no convincing evidence for the initially selfish trustee, although the associations for the two trustees did not differ significantly.

**Table 2.** Correlations between lifetime stressor exposure and computationally derived measures of trust expectation, belief updating, and negativity bias

Stress (z)	μ_0_	ω_average_	ω_pro_	ω_self_	ρ
Lifetime	−0.16^*^	0.185^*^	0.21^*^	0.13	−0.28^*^
Early life	−0.08	0.015	0.12	−0.08	−0.15
Adulthood	−0.16^*^	0.121	0.14	0.08	−0.23^*^

Note. Pearson correlations between cumulative lifetime stressor severity, as assessed by the Stress and Adversity Inventory (STRAIN), and belief parameters, derived from the HGF. Lifetime refers to all stressors experienced up to the date of the STRAIN interview, Early Life to stressors experienced prior to age 18, and Adulthood is all stressors experienced afterward. μ_0_ indexes how much reciprocity participants expected from others before receiving any evidence about them. ω refers to the expected volatility of beliefs about the interaction partner’s intentions, average, prosocial, and selfish, respectively. ρ indicates the expectation bias applied to predictions throughout the task. *P < 0.05. Boldface indicates coefficients with 95% credible intervals excluding zero.

### Higher Lifetime Stressor Exposure Is Associated with Greater Uncertainty About Others’ Intentions.

To investigate how lifetime stressor exposure affects model-implied trial-by-trial uncertainty about actors’ intentions (captured by parameter σ) in each learning phase, we ran a Bayesian MEM with σ as the dependent variable averaged across phases (learning vs. postswitch) and actor (prosocial vs. selfish). We found a robust main effect of lifetime stress (0.247, 95%CrI[0.079, 0.415]) with the posterior probability indicating a truly positive effect [P(Eμstress > 0) > 0.999], showing that greater lifetime stressor exposure was related to greater uncertainty about others’ intentions, irrespective of trustee or phase. We also found a robust double interaction for trustee × phase (0.417, 95%CrI[0.191, 0.639], P(Eμphase × trustee > 0) > 0.999). This finding indicated that changes in belief uncertainty across phases differed between prosocial and selfish trustees. Post hoc tests revealed that the behavioral switch from prosocial to selfish reduced participants’ uncertainty about the (formerly) prosocial trustee’s intentions (−0.181, CrI[−0.335, −0.023]), suggesting that violations of established trust may paradoxically produce greater confidence in negative expectations. Conversely, when the selfish trustee switched to prosocial behavior, participants showed increased uncertainty (0.236, CrI[0.080, 0.395]). Importantly, there were no robust effects for trustee × stress (0.015, 95%CrI[−0.139, 0.170]), phase × stress (−0.059, 95%CrI[−0.209, 0.098]), or the triple interaction trustee × phase × stress (0.090, 95%CrI[−0.126, 0.306]). (See Table 3 for results from the Bayesian regression.)

**Table 3.** Bayesian regression predicting uncertainty about others’ intentions (σ) from lifetime stressor exposure, trustee type, and task phase

	Estimate	low CrI	high CrI
(Intercept)	2.55	1.940	3.17
Age	−0.117	−0.256	0.023
Gender (0 = men)	−0.305	−0.638	0.025
**Phase**	**−0.181**	**−0.335**	**−0.023**
Trustee (0 = prosocial)	−0.100	−0.260	0.060
**Stress**	**0.247**	**0.079**	**0.415**
**Phase × Trustee**	**0.417**	**0.191**	**0.639**
Stress × Trustee	0.015	−0.139	0.170
Phase × Stress	−0.059	−0.209	0.098
Phase × Trustee × Stress	0.090	−0.126	0.306

Note. Fixed effects from the Bayesian regression on σ, the model-implied trial-by-trial uncertainty about others’ intentions, averaged within each phase. Age and Gender are included as covariates. For Phase, the reference group is set to the learning phase; for the interaction partner (“trustee”), the reference group is set to the prosocial trustee. Stress refers to the z-standardized cumulative lifetime stressor severity measure from the Stress and Adversity Inventory (STRAIN). Boldface indicates coefficients with 95% credible intervals excluding zero.

Together, the computational modeling results indicate that greater stressor exposure occurring over the life course is associated with a distinctive pattern of social cognition characterized by a) greater model-implied belief volatility toward the initially prosocial trustee (ω_pro_) and b) greater overall uncertainty about others’ intentions (σ), general across trustee and phase. Independent of stress, all participants became more certain following a trust violation and less certain following an improvement in trustworthiness (trustee × phase). This pattern is consistent with lifetime stressor exposure being associated with heightened sensitivity to signals of untrustworthiness, alongside less stable beliefs about a partner who has been reliable.

## Discussion

The present study investigated how cumulative lifetime stressor exposure relates to trust and dynamic trust learning. Following Social Safety Theory, we expected lifetime stress to shape the expectations that underlie trust. Using a novel approach combining a repeated trust game with behavioral reversals, computational modeling, and a comprehensive assessment of lifetime stress, we found that lifetime stressor exposure was associated with how participants invested across repeated interactions. Computational modeling indicated that greater lifetime stress was associated with lower initial trust expectations, stronger negative expectation biases, and greater belief volatility for the initially prosocial trustee, consistent with more rapid model-implied updating when a reliable partner became selfish. However, the core pattern of how people respond to trust changes remained consistent regardless of stress levels: all participants showed decreased uncertainty (“increased confidence”) after experiencing betrayal, but increased uncertainty (“heightened skepticism”) when encountering unexpected improvements in trustworthiness. This finding suggests that while stress may be associated with faster updating following negative social changes, the pattern of uncertainty changes following trustworthiness shifts appears consistent across different stress levels. Overall, these findings are consistent with lifetime stress being associated with a general pessimistic bias and heightened uncertainty about others’ intentions. While rapid belief updating in response to betrayal may serve a protective function, the broader pattern—lower initial trust and stronger negativity biases—is consistent with a shift in the expectations brought to new interactions, whose consequences for relationships and well-being will depend on the environment in which they operate.

Using a novel adaptation of the repeated trust game to include an unexpected reversal, and inferring latent beliefs with a Bayesian computational model (HGF), we show that higher lifetime stressor exposure in participants was associated with greater belief volatility (ω) for the initially prosocial trustee, with no convincing evidence for the initially selfish trustee. More volatile beliefs also mean greater trial-wise uncertainty, and hence faster updating in response to social feedback. This follows from how the model is built rather than being a separate finding. Such sensitivity may be adaptive in environments where behavior genuinely does change. These findings come from a model that accounted for initial expectations of trustworthiness (μ_0_) and a general negative expectation bias (ρ), both of which were also associated with lifetime stress. Importantly, the elevation in uncertainty about others’ intentions held regardless of partner type or task phase (Table 3), suggesting a broadly pessimistic prior rather than context-specific trust patterns. Our results suggest that higher lifetime stress may be associated with greater difficulty initiating trusting relationships with previously unknown individuals and that betrayals of trust from initially prosocial partners may have a particularly strong impact on belief updating. This interpretation aligns with evidence of detrimental effects of chronic stress on social functioning (for a review: 45). On the other hand, the increased flexibility of beliefs may also be due to a more general stress-related heightened vigilance, consistent with findings from studies using nonsocial learning tasks (23).

While this hypervigilance could be adaptive in genuinely uncertain or high-risk environments, it might lead to maladaptive behaviors in more predictable contexts, undermining the ability to foster or maintain trust. This shift in attention broadly aligns with research that has shown that acute stress—and associated uncertainty—can lead to a shift in attention chiefly by increasing threat detection (40) and in particular, by biasing social attention toward negative social information (37, 39, 46, 47). While research on more perpetual or recurring forms of stress is limited, there is evidence that perpetual states of uncertainty—such as anxiety (48, 49) or loneliness (50, 51)—can lead to similar biases in information processing. Furthermore, it has been suggested that lifetime experiences shape expectations and behavior, including in social contexts (52). As such, lifetime stressors may reflect, or relate to, a recalibration of social threat assessments (2), and correspondingly, differential expectations about how others will interact with oneself. Of note, lower levels of initial trust may serve as a protective mechanism, allowing to minimize vulnerability in potentially harmful situations. However, this bias could also limit opportunities to form positive relationships, which might deter engagement with new social partners. In particular, childhood stressors have been linked to different relationship schemas (or expectations about social interactions more generally). While early life stress has been related to social behaviors and expectations (53–55), our findings suggest that cumulative lifetime stressor exposure—encompassing both childhood and adulthood experiences—was most consistently associated with our computationally derived parameters of trust expectations and belief updating.

Our data also show that greater cumulative lifetime stressor exposure is associated with stronger differentiation between prosocial and selfish trustees. Specifically, higher levels of lifetime stress were associated with reduced investments in selfish, but not prosocial, trustees. This heightened differentiation may reflect an adaptive response to chronic adversity, where prioritizing caution toward potentially harmful actors might take precedence (56). This finding is broadly consistent with evidence from the acute stress literature, which has shown that stress is associated with a shift from more deliberate to more automatic, bottom–up information processing (for reviews: 40, 42), potentially enabling more reflexive responses to social threats.

Crucially, we found that more lifetime stressor exposure was associated with greater overall uncertainty (σ) about both trustee types, independent of experimental phase or trustworthiness reversals. The volatility parameter (ω) showed the association with stress for the initially prosocial trustee, though this did not differ significantly from the selfish trustee. Although prior research has shown that negative moral impressions naturally elicit greater belief volatility (57), our findings are consistent with greater lifetime stress being associated with a cognitive state in which positive impressions are held with less certainty (“Are they really that good?”). This higher uncertainty about good actors makes beliefs more malleable, allowing for faster detection when trust is violated. Whether this profile is adaptive or costly likely depends on the context, as work on adversity-shaped cognition has argued (58): Sensitivity to volatility may be protective when partners are unreliable and costly when they are dependable. Notably, this profile was not accompanied by poorer differentiation between partners—higher lifetime stress was associated with reduced investment in the selfish trustee and comparable investment in the prosocial one (Table 1). Lifetime stress is thus associated with a shift in what is expected and how firmly it is held, rather than with a failure to read partners accurately, a pattern consistent with a reweighting of prior belief against incoming evidence, whose costs and benefits depend on the environment in which it operates.

This pattern aligns with research showing that chronic stress is associated with reduced perceptions of environmental stability (23) and increased avoidance of ambiguous situations (32). Importantly, the volatility association was present for the initially trustworthy partner, while the elevation in uncertainty applied across partners and phases. Less stable expectations about otherwise reliable partners may be one route by which lifetime stress is associated with social withdrawal and difficulties maintaining cooperative relationships (1, 59), despite preserved ability to differentiate between trustee types. An important limitation of our study is that the volatility introduced in our experimental design was relatively abrupt. Future research should employ designs with more gradual and systematic manipulations of social volatility to better capture real-world dynamics.

### Limitations and Future Directions.

Several limitations of this research need to be considered when drawing conclusions from this work. First, the design is cross-sectional and correlational: Lifetime stressor exposure was assessed retrospectively using a structured interview and was not manipulated, so we cannot infer directionality or causation. Although the STRAIN’s branching interview format and psychometric validation reduce recall concerns relative to brief checklists, retrospective reports of stress can be influenced by unmeasured third variables (e.g., negative affect, current distress). The associations we report are also modest in size. Because lifetime stress cannot be randomly assigned, causal inference will require designs that approximate rather than impose it, such as longitudinal or natural-experiment approaches, alongside experimental work on acute stress. We interpret our findings as associations and as a first step, motivating this work. Second, our controlled laboratory paradigm—while carefully designed to balance experimental rigor with ecological validity—may not fully capture the nuanced complexity of real-world trust and social interactions. Future studies could build on these findings through naturalistic observations, longitudinal field designs, or experience sampling methodologies to better reflect everyday trust dynamics. Third, our operationalization of stress as cumulative exposure to major life stressors, though broadly applicable, represents just one dimension of this multifaceted construct. Future research could examine less severe stressors, as well as psychological stress reactivity (rather than exposure) and biological stress markers (e.g., cortisol, inflammation), to provide a more comprehensive understanding. Finally, our sample was largely composed of young, healthy adults, which may limit generalizability across age groups, cultural contexts, and clinical populations. More diverse samples with greater variation in stress exposure and sociodemographic backgrounds are needed to test the robustness and boundaries of these effects.

## Conclusion

The present data are among the first that we know of to characterize how cumulative lifetime stress is associated with the cognitive architecture of trust learning—a cornerstone of human social interaction—and to suggest a profile consistent with recalibration rather than degradation. Through a combination of computational modeling and behavioral experimentation, we find that greater lifetime stressor exposure is associated with less initial trust, stronger negativity biases, and greater belief volatility when evaluating others’ intentions. These more pessimistic expectations were not accompanied by poorer reading of partners, as higher lifetime stress was associated with greater differentiation between prosocial and selfish trustees. This pattern is consistent with a profile that is both more sensitive to potential betrayals and more cautious in forming new social connections. Our findings also show a distinct cognitive pattern associated with lifetime stress across trust formation and adaptation. The heightened uncertainty we observe was general across partners and phases, while the association with belief volatility was carried by the initially prosocial trustee. Together, these suggest that lifetime stress is associated not only with differences in social functioning but with differences in the processing of social information and subsequent behavior (60, 61). Such a profile may be adaptive in unpredictable environments, yet may carry costs where it destabilizes the relationships that typically buffer against stress.

Given that social relationships are among the strongest predictors of health and longevity (8–10, 62, 63), our results are consistent with the possibility that lower trust, poorer relationship quality, and reduced access to social support are mutually associated, a configuration that could heighten vulnerability to future stressors. The computational approach developed here advances our understanding of associations between adversity and social cognition, while generating testable predictions about when these stress-related cognitive adaptations become maladaptive.

## Materials and Methods

### Participants.

Participants were recruited through the Vienna CogSciHub Study Participant Platform using hRoot software (64). From an initial sample of 170 participants, two individuals were excluded due to invariant behavior during the learning phase of the decision-making game, indicating noncompliance. The final sample comprised 168 participants (Age: Mean = 26.7 SD = 8.09, range = 18 to 68; 38 men).

We targeted 150 participants (sensitivity analysis for r = 0.25, α = 0.05, two-tailed) and oversampled to 170 to allow for exclusions (*SI Appendix*). The study was approved by the University of Vienna Research Ethics Board and conducted in accordance with the Declaration of Helsinki. Participants received 22 Euros in compensation: a 20 Euro base payment for participation and 2 Euros for the trust game (i.e., all participants, regardless of performance, received the maximum payout).

### Procedure.

Participants arrived in groups (group size: mean = 5.2; SD = 1.7) at the laboratory and gave written consent for participation. Following this, the participants were introduced to a “social interaction task” (i.e., the repeated trust game, see below) that they would be playing with other participants, some of whom would connect remotely. In reality, the behavior of all coplayers was determined by programmed computer algorithms. Participants were told to carefully read the instructions before the experimenter started the task to allow for synchronized participation of all participants. Afterward, participants completed several questionnaires (*SI Appendix*), including a demographics survey and an inventory to assess lifetime stressor exposure (see below). Following the task, participants completed a funnel debriefing; 42 (25%) correctly identified the deception. Control analyses including deception and group size as covariates did not change the results (*SI Appendix*, Table S4).

### Lifetime Stressor Exposure.

To measure lifetime stressor exposure, we used the Stress and Adversity Inventory for Adults (STRAIN) (43), administered in its validated German-language version (44). The STRAIN is an online, semistructured, automated interview that assesses stressors occurring across 12 major life domains (e.g., housing, education, work, health, partner, reproduction, financial, legal, and life-threatening situations) and five social-psychological characteristics (i.e., interpersonal loss, physical danger, humiliation, entrapment, role change/disruption) and 55 types of major acute and chronic stressors that may have occurred over the participant’s lifetime (see http://www.strainsetup.com). Questions are presented in a branching fashion; for each stressor that participants endorsed, they are asked a series of follow-up questions. For all analyses, we focused on the STRAIN’s assessment of cumulative lifetime stressor severity, which was a continuous variable. We also separately examined the effects of stressors occurring in early life vs. adulthood to probe for potential differences by stress exposure timing. The STRAIN has excellent psychometric properties (43, 44) and has been used in over 70 peer-reviewed publications to date, including studies that have assessed the impact of lifetime stressor exposure on reward processing, learning, and decision-making (32, 34–36).

### Trust and Trust-Learning.

Real-world trust is rarely static—it evolves through repeated interactions and must adapt to sudden changes in others’ behavior. To capture this dynamic and adaptive nature of trust, we developed a modified repeated trust game (18–20, 65). In this paradigm, participants (Player A; investors) were endowed with 10 points (credits) per trial and decided how many to transfer to a trustee (Player B; simulated by a computer algorithm). Transferred points were tripled, and the trustee then decided how many points to return. Participants interacted with two trustees in an interleaved design: a prosocial trustee and a selfish trustee. To establish baseline trust, both trustees returned 100% of the investment in the initial two trials. For trials 3 to 34 (i.e., 16 per trustee), the trustees’ behaviors were designed to simulate naturalistic social interactions with inherent variability and adaptability. The prosocial trustee responded with probabilistic variability to mimic realistic cooperative behavior: returning the initial investment at 100%, returning 150% of the initial investment, or returning double the initial investment (200%). Similarly, the selfish trustee exhibited naturalistic uncooperative behavior: returning the initial investment at 100%, returning 75% of the initial investment, or returning 50% of the initial investment. All returned amounts were integers, rounded down to the nearest whole number. For example, if Player A invested 5 points (tripled to 15), the prosocial trustee could return 5 (100%), 7 (150%), or 10 (200%) points, while the selfish trustee could return 5 (100%), 4 (75%), or 3 (50%) points.

To further enhance the naturalistic quality of the interactions, the probability matrix for each trustee’s behavior was dynamically adjusted based on participant responses. This adaptive mechanism meant that if participants increased their sharing to the prosocial trustee, the likelihood of the prosocial trustee returning the most generous response (200%) increased by 10% in the next interaction. Conversely, if participants decreased their sharing to the selfish trustee, the likelihood of the selfish trustee returning the least generous response (50%) increased by 10% in the next interaction (19, 65). This adaptive design allowed the trustees’ behaviors to evolve in response to the participants’ trust decisions, creating a more realistic and dynamic social interaction. After trial 34, the trustees’ behaviors switched, with the prosocial trustee becoming selfish and vice versa, adopting only the most extreme responses (200% and 50%, respectively). This reversal phase (trials 35 to 50) allowed us to investigate how participants adapted their trust decisions to sudden changes in trustee behavior. The design thus enabled examination of both initial trust formation and adaptation to behavioral reversals. To enhance ecological validity, participants were informed they were interacting with real people via remote connection. The task was implemented in Python using PsychoPy (66).

## Analyses

### Behavioral Analysis.

Behavioral analysis was conducted with Bayesian multilevel (generalized) ordinal logistic regression (67), fitted with the “brms” package in R (68, 69). All models were run with uninformed priors (70) (Normal distribution, mean = 5, SD = 5 for Intercept; mean = 0, SD = 2 for predictors, and half Cauchy, mean = 0, SD = 2 for error terms). All models were run with four chains with 4,000 iterations, each with 1,000 warmups. We visually inspected the quality of chain convergence based on trace plots of main fixed effects, and we set the threshold on the Gelman–Rubin R̂ Statistic for each parameter to 1.01 (71). The confidence of whether or not an effect was present is reflected by the 95% Credibility Interval (CrI) (72), as well as the posterior probability that a certain effect (*Eμ*) is different from 0 [*P*(*Eμ* < 0) or *P*(*Eμ* > 0)].

We propose that there is compelling evidence for an effect if its posterior probability is close to *Eμ* = 1 (73). Therefore, we ran a mixed-effects model with rank-ordered investment amounts (family = cumulative) as the dependent variable, and trustee (0 = prosocial actor; 1 = selfish actor), interaction number (1 to 17 for each actor; 34 trials), and STRAIN scores for severity as the predictor variables. Participant ID was entered as a random intercept for the random effects, nested within the double interaction of the trial number and interaction partner for a maximally defined random effect (74, 75). As we were interested in the effects of stress on differential behaviors for each trustee, we added all double interactions for these variables (i.e., trustee, trials, stress). In addition, we included age (z-scored) and gender (men coded as the reference group) of the participants as covariates. Post hoc tests were run on the model-based posteriors using the hypothesis function from the brms package.

### Computational Modeling.

We used the Hierarchical Gaussian Filter (HGF) (21, 22) to estimate each participant’s learning characteristics and belief trajectories during the repeated trust game, including the reversals. The HGF has been extensively used to model how agents form beliefs, in a hierarchical fashion, and about hidden states in the environment. The belief model is coupled with a response model that specifies how belief estimates map onto decisions. HGF parameter estimation was carried out using custom and previously validated (20) code in the programming language Stan version 2.33 (76). Participants were fitted separately, and all models were run with four chains with 4,000 iterations, each with 1,000 warmups. All models converged, with Gelman–Rubin R̂ below 1.01 for all parameters (71). Notably, the data used for parameter estimation included the entire experiment, including the switch in behavior. As such, the derived parameters indicate expectations regarding the two trustees’ behaviors in a (relatively) volatile environment. Four different versions of the HGF model and two versions of the Rescorla–Wagner model were compared (*SI Appendix*) with Leave-One-Out Cross-Validation (LOO-CV) using the *loo* package in R (77). The winning model uses five parameters, plus a response-noise parameter (η): initial reciprocity expectation (μ_0_) and its uncertainty (σ_0_) at the first trial; an expectation bias (ρ) capturing a positive prior toward reciprocation; and two volatility parameters estimated separately for the prosocial and selfish trustee (ω_pro_, ω_self_), with higher values indicating more volatile beliefs. The volatility parameters govern the trial-by-trial uncertainty about others’ intentions, σ(t), which sets the rate of belief updating. Full model equations and parameter definitions are provided in *SI Appendix*, Table S2 a and b. While μ_0_ indicates expectations at the onset of the experiment, we averaged the trial-by-trial uncertainty σ(t) within each of two phases (learning, postswitch) to investigate the model-implied uncertainty about others’ intentions across the task. An exploratory analysis of overlap among these parameters is reported in *SI Appendix*, Table S6. Following modeling and parameter determination, we then correlated the μ_0,_ ρ, ω_pro_, and ω_self_ parameters with the overall severity scale from the STRAIN, as well as the subscales for early life stress severity and adult life stress severity. Where two dependent correlations sharing a common variable were compared, we used the Williams test (78).

Next, we ran a Bayesian Mixed Effects model with σ(t) as the dependent variable to test the effect of stress on uncertainty about others’ intentions and to test for phase-specific effects. Here, we aggregated σ_ts_ for each of the two phases (learning, postswitch) by trustee to test if participants had differential levels of uncertainty for the type of trustee (prosocial vs. selfish), by phase (learning vs. postswitch), and for varying levels of lifetime stress (continuous variable). Gender and age (z-scored) were added as covariates. Since we were interested in the differential effect of lifetime stress on decision-making for the selfish and prosocial trustee based on phase, we added interactions for each of the main effects, trustee × phase, trustee × stress, stress × phase, as well as the triple interaction trustee × phase × stress. Participant ID was nested within the additive slope of phase and trustee for the random effect to facilitate the MC sampling.

## Supplementary Material

Appendix 01 (PDF)

## Data Availability

Anonymized data, analysis scripts, and the Stan model code are available on the Open Science Framework (https://osf.io/xtzeq/) (79). All other data are included in the manuscript and/or *SI Appendix*.
