# Supplementary material for: Lifetime stressor exposure is associated with altered trust formation and social learning in dynamic interactions
Source: Proc Natl Acad Sci U S A. 2026 Nov 2;123(45):e2608572123. doi: 10.1073/pnas.2608572123 (PMC13752333; doi:10.1073/pnas.2608572123)
Supplement: Supplementary file 1 [file pnas.2608572123.sapp.pdf]

Supplemental Information Appendix

Lifetime stressor exposure is associated with altered trust formation and social learning in dynamic interactions.

Content

1. Sample and Power Considerations
2. All intercepts of the Bayesian Regression Model
3. Computational Modelling
4. Model comparisons
5. Regression with Deception and Group-Size
6. Exploratory decomposition of stress-related associations
7. Correlations among stress-related parameters
8. Sensitivity analyses: feedback dependence and initial investments
9. Survey and Questionnaire Materials
10. Artificial Intelligence Disclosure

1. **Sample and Power Considerations**

Sample:

Participants comprised a nonprobability convenience sample recruited from the University of Vienna participant pool using the Vienna CogSciHub Study Participant Platform (hRoot). Recruitment was based on voluntary enrollment rather than probability-based sampling procedures. Consequently, the sample should not be considered representative of the broader Austrian population. The final sample included 168 adults (mean age = 26.7 years, SD = 8.09, range = 18-68 years; 38 men) after exclusion of two participants who showed invariant behaviour during the learning phase of the task. Data collection was conducted between 31.01.2023 and 11.05.2023 at the Department of Cognition, Emotion, and Methods in Psychology, University of Vienna.

Power:

Given our goal to generate hypotheses about the relationship between cumulative lifetime stress and dynamic trust processes, an area with limited prior empirical work, we targeted a sample size of 150 participants. This target was informed by a sensitivity analysis for our primary association ($r = 0.25$, $\alpha = 0.05$, two-tailed), which indicated adequate sensitivity to detect an effect of this size. To account for potential dropouts and imprecision in effect-size estimates, we oversampled to 170 participants; the final sample was 168 after two exclusions for invariant behaviour during the learning phase. Given the exploratory, hypothesis-generating aims of the study, we treat the analyses as estimates of associations rather than confirmatory tests and interpret the effects accordingly.

2. **All intercepts of the Bayesian Regression Model**

Supplementary Table S1: Intercepts for Bayesian Regression

term	estimate	conf.low	conf.high
(Intercept)[1]	-11.0	-12.4	-9.64
(Intercept)[2]	-3.69	-4.13	-2.64
(Intercept)[3]	-2.84	-3.89	-1.79
(Intercept)[4]	-2.18	-3.23	-1.13
(Intercept)[5]	-1.59	-2.64	-0.544
(Intercept)[6]	-0.685	-1.73	0.357
(Intercept)[7]	-0.137	-1.18	0.908
(Intercept)[8]	0.426	-0.611	1.48
(Intercept)[9]	1.15	0.108	2.20
(Intercept)[10]	1.79	0.753	2.84

Note. Intercepts from the Rank-ordered Bayesian regression on investment amount (Table 1 in Manuscript).

3. Computational Modelling

Model Description

The HGF infers beliefs via update equations for each agent, given the free parameters (e.g., ω, ρ). Initial reciprocity expectation (μ_0) and its uncertainty (σ_0) are defined at the first trial, are uninformed by feedback, and are assumed identical for both interaction partners. The expectation bias parameter (ρ) captures a positive prior toward reciprocation. Two volatility parameters are estimated separately for the prosocial and selfish trustee (ω_{pro}, ω_{self}), with higher values indicating more volatile (less stable) beliefs about the trustee's intentions; we denote their per-participant average as ω_average. The volatility parameters govern the evolution of the trial-wise uncertainty about others' intentions, $\sigma(t)$, which in turn sets the rate of belief updating (the learning rate). Full update equations are given in Tables S2a–b; the equations shown are indexed for the prosocial partner (pro) and are identical for the selfish partner, and parameters without a pro/self index are shared across partners.

Supplementary Table S2a. Equations for Computational Parameter

**Inferred beliefs**	$x^{(t)} \sim N\left(\mu^{(t)}, \sigma^{(t)}\right)$
Prediction error	$PE = \mathrm{BT(t)} - \mu_{\mathrm{pro,1}}(\mathrm{t}-1)$
Update Equations	$\sigma_{\mathrm{pro,2}}(\mathrm{t}) = \dfrac{1}{\dfrac{1}{\sigma_{\mathrm{pro,2}}(t-1) + \exp\left(\omega_{\mathrm{pro}}\right)} + \sigma_{\mathrm{pro,1}}(\mathrm{t}-1)}$
	$\mu_{\mathrm{pro,2}}(t) = \mu_{\mathrm{pro,2}}(\mathrm{t}-1) + \sigma_{\mathrm{pro,2}}(t) \cdot PE + \rho$
	$\mu_{\mathrm{pro,1}}(\mathrm{t}) = \dfrac{1}{1 + \exp\left(-(\mu_{\mathrm{pro,2}}(\mathrm{t}) + \rho\right)}$
	$\sigma_{\mathrm{pro,1}}(t) = \mu_{\mathrm{pro,1}}(\mathrm{t})\left(1 - \mu_{\mathrm{pro,1}}(\mathrm{t})\right)$
Response Model	$\alpha = \nu\mu_{\mathrm{pro,1}}(\mathrm{t})$
	$\beta = \nu\left(1 - \mu_{\mathrm{pro,1}}(\mathrm{t})\right)$
	$\mathrm{Investment(t)} \sim \mathrm{Beta}(\alpha, \beta)$

Supplementary Table S2b. Glossary for Trajectories

$\mu_{\mathrm{pro,2}}$ $(\sigma_{pro,2})$	beliefs (uncertainty) about trustworthiness in continuous space
$\mu_{\mathrm{pro,1}}$ $(\sigma_{pro,1})$	beliefs (uncertainty) about trustworthiness in contingency space
**BT**	back transfer

Parameters

$\mu_{\mathrm{pro,2}}(0)$ $(\sigma_{pro,2}$	Initial trustworthiness belief (uncertainty)

(0))

ω_{pro}	Volatility of beliefs about trustworthiness
ρ	Expectation bias (a positive number denotes higher expectation of reciprocation)

Note: Glossary for Trajectories

4. Model Comparison

We compared four different HGF models and two Rescorla-Wagner models.

Table S3.

**Label**	**Parameters**	**ELPD (mean)**	**ELPD (SE)**
HGF 4	ω_self, ω_pro, μ_0, σ_0, ρ, \eta	3363	251
RW 1	\alpha_pos, \alpha_neg ,\beta,v_0	3016	255
RW 2	\alpha_pos, \alpha_neg\beta, fixed: v_0	2464	253
HGF 3	ω_self, ω_pro, μ_0, σ_0, ν, fixed: ρ	2247	225
HGF 1	ω_self, ω_pro, σ_0, ν, ρ μ0 fixed	1921	220

HGF 2	ω_self, ω_pro, , ν<br><br>fixed ρ μ_0, σ_0	1279	211

Supplementary Figure S1

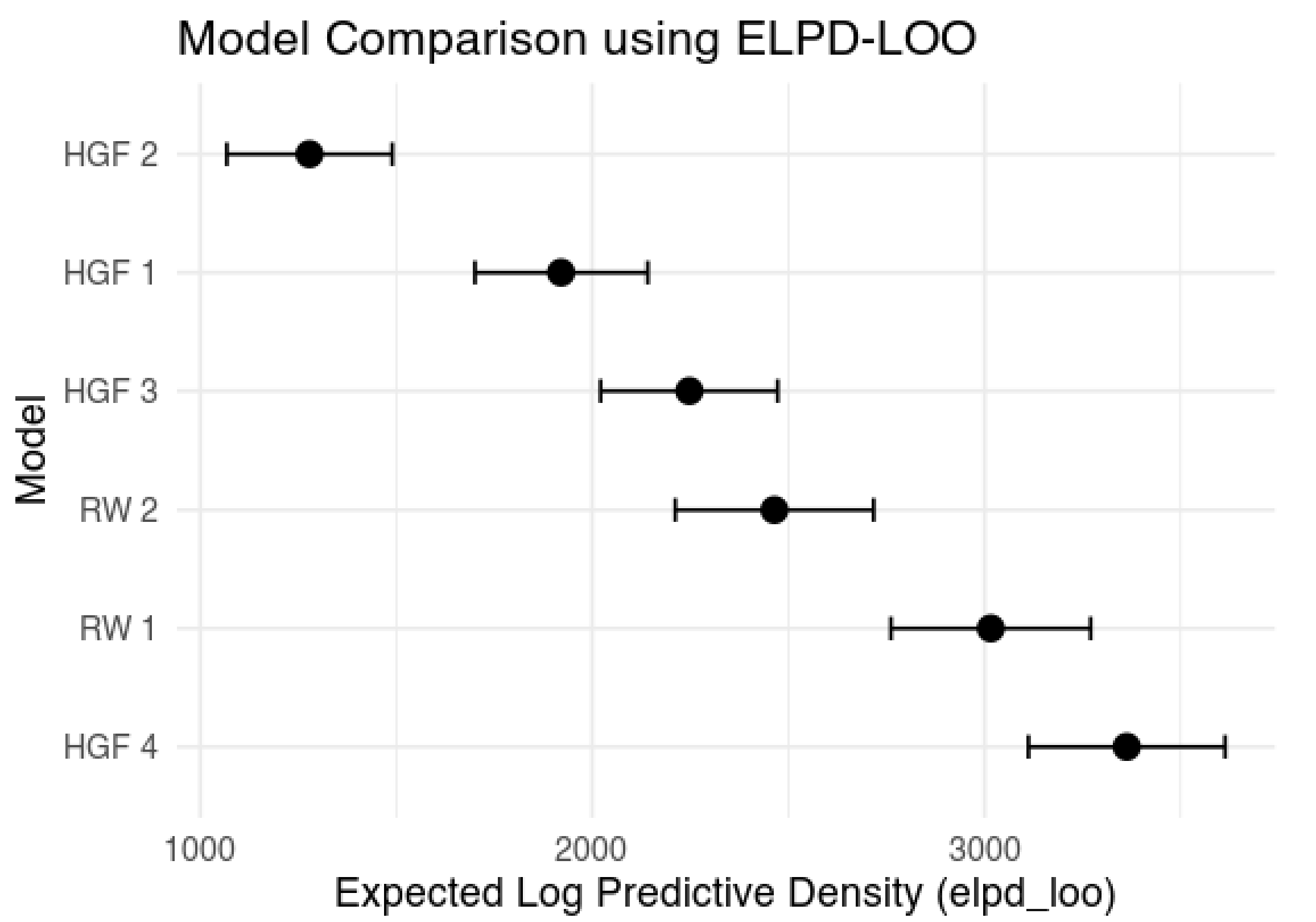

Figure S1. The plot compares the fit of four Hierarchical Gaussian Filter (HGF) models and two Rescorla-Wagner models in explaining participants' trust formation and social learning. Higher ELPD values indicate better model performance. The HGF models incorporate parameters for initial trustworthiness belief, volatility, expectation bias, and response noise, while the Rescorla-Wagner models serve as simpler benchmarks. The comparison highlights the relative explanatory power of each model in capturing behavioural dynamics during the trust task.

5. **Regression Model Including Deception and Group Size as Covariates**

Participants completed the following debriefing questionnaire at the conclusion of the study:

1. Did you have any doubts about the authenticity of the experimental setup?

Response options: Yes/No

If you had doubts, please describe them:

Open-ended response.

2. Please describe what you believe this study was investigating:

Open-ended response.

3. Do you have any additional comments regarding how the procedure could be improved or made more pleasant for participants?

Open-ended response.

Participants who had doubts and could correctly name the deception (i.e., "computer players") with any confidence (including low) were labelled with a 1; everyone else was labelled with a 0. Forty-two participants (25%) reported a deception (of those, 35 with low confidence and 7 with moderate to high confidence).

We also included group size—how many participants were tested simultaneously—as a covariate in the analysis. Note that running two separate models instead of one results in similar results. Importantly, even when including these covariates, the effect of the "Stress✕Interaction Partner" interaction remains meaningful.

Supplementary Table S4: Rank-ordered Bayesian regression on investment amount, with deception and group size as covariates.

	**Estimate**	**low CrI**	**high CrI**
Intercepts	*omitted*		
Age (z)	0.03	-0.21	0.26
Gender	-0.094	-0.628	0.436
Deception (0= no deception)	0.10	-0.16	0.33
Group-size	-0.06	-0.19	0.07
Trial	0.194	0.164	0.224

Trustee	0.034	-0.209	0.273
Stress	-0.209	-0.508	0.088
Trial×Trustee	**-0.399**	**-0.439**	**-0.359**
Stress×Trustee	**-0.285**	**-0.479**	**-0.086**
Trial×Stress	-0.004	-0.027	0.019

Note. Fixed effects from the ordinal Bayesian regression on investment amount (rank-ordered; 1-10). Age and Gender are included as covariates. For the interaction partner ("trustee"), the reference group is set to the prosocial trustee. Stress refers to the z-standardized cumulative lifetime stressor severity measure from the STRAIN. The ten intercepts from the ordinal model are not included in the table for clarity.

6. Exploratory decomposition of stress-related associations

To assess whether stress is associated with model-implied initial trust (μ_0) overlap with those for belief volatility (ω) and expectation bias (ρ), we conducted an exploratory Bayesian decomposition in brms. Belief volatility entered as ω_average, the per-participant mean of ω_pro and ω_self. The three regression equations (stress → ω_average; stress → ρ; μ_0 ~ stress + ω_average + ρ) were fitted jointly without residual correlations, and shared components were computed as the product of the corresponding posterior draws. Note that μ_0 is defined before the first trial, whereas ω governs belief updating across trials. Because all parameters derive from the same behavioural data and the design provides no temporal ordering among them, these estimates describe concurrent overlap rather than directional pathways.

Negativity bias was robustly associated with initial trust (b = 0.23, 95% CrI [0.07, 0.39]), whereas the association between belief volatility and initial trust did not reach the same level of credibility (b = 0.14 [−0.01, 0.30]).

The component shared between stress, negativity bias, and initial trust was robust and negative (b = −0.063 [−0.129, −0.015], P(< 0) = .997). The corresponding component for

belief volatility was not credible (b = 0.026 [−0.003, 0.073]) and ran opposite in sign. The total shared component across both parameters did not clearly exclude zero (b = −0.037 [−0.105, 0.024]). Accounting for both, the direct association between stress and initial trust was attenuated relative to the bivariate association reported above and no longer fully credible (b = −0.124 [−0.279, 0.029], P(< 0) = .944), while the total association remained robust (b = −0.161 [−0.317, −0.008], P(< 0) = .981).

Supplementary Table S5. Exploratory decomposition: direct, shared, and total associations of lifetime stress with initial trust (μ_0).

Path	Estimate	low CrI	High CrI	P(< 0)
direct: stress → μ_0	−0.124	−0.279	0.029	.944
shared: stress, ρ, μ_0	−0.063	−0.129	−0.015	.997
shared: stress, ω, μ_0	0.026	−0.003	0.073	.047
shared: total	−0.037	−0.105	0.024	.882
total: direct + shared	−0.161	−0.317	−0.008	.981

Note. Posterior medians with 95% credibility intervals. P(< 0) is the posterior probability that the effect is negative. Shared components computed as products of posterior draws.

7. Correlations among stress-related parameters

To assess whether the stress-related parameters reflect distinct or overlapping components, we examined their correlations with one another, both in their raw form and after removing shared stress-related variance. For the latter, we regressed μ_0, ρ, and ω_pro (z-scored) on lifetime stress, age, and gender in a single multivariate Gaussian model (brms; residual correlations estimated with set_rescor = TRUE; priors: Normal(0, 2); 4 chains × 4000 iterations; n = 167). We did not include the trial-level uncertainty σ, since the volatility parameters govern the evolution of $\sigma(t)$ and the two are therefore coupled by construction rather than independently estimated; the three parameters entered here are separately estimated free parameters of the model. ρ and ω_pro were uncorrelated, both in the raw data

(r = −0.06 [−0.21, 0.09]) and after partialling (r = −0.00 [−0.15, 0.15]), indicating that the negativity bias and belief volatility are separable components. Both showed modest correlations with μ_0, again similar raw and residual (Table S6). All parameters converged ($\hat{R}$ = 1.00, bulk-ESS > 16,000).

Supplementary Table S6. Correlations (95% CrI) among μ_0, ρ, and ω_pro

	ρ	μ_0	ω_pro
ρ	-	0.18 [0.03, 0.32]	−0.00 [−0.15, 0.15]
μ_0	0.21 [0.06, 0.35]	-	0.17 [0.01, 0.32]
ω_pro	−0.06 [−0.21, 0.09]	0.14 [−0.01, 0.29]	-

Note. Below diagonal: raw Pearson correlations with 95% confidence intervals (n = 168). Above diagonal: residual correlations with 95% credibility intervals, from a three-outcome multivariate model regressing z-scored μ_0, ρ, and ω_pro on lifetime stress, age, and gender (n = 167; one participant excluded for missing covariate data).

8. Sensitivity analyses: feedback dependence and initial investments

Because trustee return probabilities are contingent on participants' investments, we ran two sensitivity analyses to assess whether the association between lifetime stressor exposure and differential investment could be attributed to differences in the feedback participants received.

Both models used the same predictors, trial window (trials 1–17), priors, and sampling settings as the primary investment model reported in Table 1 (brms, cumulative family, 4 chains, 4,000 iterations, 1,000 warmup, adapt_delta = 0.9999, max_treedepth = 12).

We first estimated whether the returns participants received varied with lifetime stressor exposure, using realized returns as the outcome rather than investment. The stress × trustee interaction was not distinguishable from zero (β = −0.24, 95% CrI [−0.51, 0.04], P(β < 0) = .95).

We then re-estimated the primary investment model, including each participant's realized return from the same trustee on the preceding trial as a trial-level covariate. Previous-trial feedback predicted subsequent investment (β = 0.21, 95% CrI [0.19, 0.23]), and the stress × trustee interaction remained credible (β = −0.39, 95% CrI [−0.63, −0.14]). Trial-1 observations are excluded from this model, as no preceding trial is available (n = 5,344, vs. n = 5,678 above).

All parameters converged ($\hat{R} \leq 1.01$, bulk-ESS > 1,000).

Table S7: Sensitivity analyses: feedback dependence

Parameter	Feedback: Estimate	Feedback: low CrI	Feedback: high CrI	Adjusted: Estimate	Adjusted: low CrI	Adjusted: high CrI
Age	−0.03	−0.20	0.15	0.07	−0.12	0.25
Gender (0 = men)	0.17	−0.17	0.52	0.20	−0.18	0.59
Trial	0.25	0.22	0.28	0.06	0.03	0.08
Trustee (0 = prosocial)	−1.30	−1.59	−1.02	0.23	−0.01	0.47
Stress	−0.13	−0.41	0.16	−0.16	−0.41	0.08
Previous-trial return	—	—	—	0.21	0.19	0.23
Trial × Trustee	−0.54	−0.59	−0.50	−0.21	−0.25	−0.18
Stress × Trustee	−0.24	−0.51	0.04	−0.39	−0.63	−0.14
Trial × Stress	0.01	−0.02	0.03	−0.01	−0.03	0.01
Trial × Stress × Trustee	−0.02	−0.07	0.03	0.01	−0.02	0.05

Note. Fixed effects from ordinal Bayesian regressions. Intercepts omitted for clarity. The left model predicts the return received from each trustee; the right predicts investment, adjusting for the return received on the preceding trial from the same trustee.

Correlations. Lifetime stressor exposure was associated with participants' initial investments ($r = -0.20$, 95% CI [−0.34, −0.05], $p = .009$, $n = 167$). Initial investments correlated with μ_0 ($r = 0.87$, 95% CI [0.83, 0.90], $p < .001$, $n = 168$).

9. Survey and Questionnaire Materials

Demographics items

Participants completed a demographic questionnaire in German via Qualtrics. Items assessed gender, hormonal contraceptive use, menstrual-cycle characteristics, age, educational attainment, field of study, and perceived financial situation. Only age and gender were used in the present analyses.

- „Was ist Ihr Geschlecht?" [What is your gender?] Response options: [male/ female/ other].
- „Was ist Ihr Alter? (Ziffern eintragen)" [What is your age? (enter digits)] Open numeric response.

Lifetime Stressor Exposure Assessment

Lifetime Stressor Exposure (Adult STRAIN). Lifetime stressor exposure was assessed using the German-language version (Sturmbauer et al., 2019) of the Stress and Adversity Inventory for Adults (Adult STRAIN; Slavich & Shields, 2018). The STRAIN is an online, automated interview administered on a licensed platform separate from the Qualtrics questionnaire battery. Stressor scores were generated automatically by the STRAIN system.

Structure. The interview assesses 55 major stressors, comprising 26 acute life events and 29 chronic difficulties. These stressors span 12 primary life domains (housing, education, work, treatment/health, marital/partner, reproduction, financial, legal/crime, other relationships, death, life-threatening situations, and possessions) and are additionally classified according to five core social-psychological characteristics: interpersonal loss, physical danger, humiliation, entrapment, and role change/disruption (Table S8).

Administration and response format. Questions are presented one at a time. Participants first indicate whether they have experienced a given stressor. For endorsed stressors, follow-up questions assess characteristics such as perceived severity, frequency, timing, and duration. Because these follow-up questions depend on previous responses, the

number, content, and order of interview items vary across participants. Consequently, there is no fixed item set or administration sequence that applies to all respondents.

Scoring. The present analyses focused on cumulative lifetime stressor severity, defined as the summed perceived severity of all endorsed stressors. Corresponding severity scores for stressors occurring during early life (<18 years) and adulthood were also examined.

Availability. The Adult STRAIN is a licensed instrument; therefore, its complete item bank, branching logic, and follow-up item wording cannot be reproduced here. Information regarding instrument access, development, and validation is available in Slavich and Shields (2018), Sturmbauer et al. (2019), and at https://www.strainsetup.com.

Table S8. Dimensions of lifetime stress assessed by the Adult STRAIN (adapted from Slavich & Shields, 2018).

Dimension	Categories
Exposure indices	Stressor count; stressor severity (used here)
Exposure timing	Early life (<18 years); adulthood
Stressor types	Acute life events (26); chronic difficulties (29)
Primary life domains (12)	Housing; education; work; treatment/health; marital/partner; reproduction; financial; legal/crime; other relationships; death; life-threatening situations; possessions
Core social-psychological characteristics (5)	Interpersonal loss; physical danger; humiliation; entrapment; role change/disruption

Additional Task

Following completion of the Adult STRAIN, participants completed an Emotion Perception and Recognition Task. In this computerized task, participants rated and identified facial expressions of varying emotional intensity using morphed stimuli from the FACES database (Ebner et al., 2010). Data from this task were not analyzed in the present manuscript and will be reported separately.

Order of procedures

Participants completed study procedures in the following order:

1. Informed consent
2. Instructions for the social interaction task
3. Repeated trust game
4. Online questionnaire battery (Qualtrics), in the following order: UCLA Loneliness Scale; Childhood Trauma Questionnaire (short form); Interpersonal Reactivity Index; Social Value Orientation slider measure; Chalder Fatigue Scale; Liebowitz Social Anxiety Scale; Perceived Stress Scale (10-item); physical health symptoms questionnaire; Social Network Index; PANAS (short form); demographic items (see above). Apart from age and gender, these measures were not analyzed in the present manuscript.
5. Stress and Adversity Inventory (STRAIN)
6. Emotion Perception Task
7. Funnel debriefing (items reproduced in the section *Regression Model Including Deception and Group Size*)

10. Artificial Intelligence Disclosure

No artificial intelligence (AI) tools were used in data collection, data preprocessing, statistical analysis, computational modelling, or interpretation of the results presented in this manuscript.
